# Biallelic variants in *PCDHGC4* cause a novel neurodevelopmental syndrome with progressive microcephaly, seizures, and joint anomalies

**DOI:** 10.1038/s41436-021-01260-4

**Published:** 2021-07-09

**Authors:** Maria Iqbal, Reza Maroofian, Büşranur Çavdarlı, Florence Riccardi, Michael Field, Siddharth Banka, Dalal K. Bubshait, Yun Li, Jozef Hertecant, Shahid Mahmood Baig, David Dyment, Stephanie Efthymiou, Uzma Abdullah, Ehtisham Ul Haq Makhdoom, Zafar Ali, Tobias Scherf de Almeida, Florence Molinari, Cécile Mignon-Ravix, Brigitte Chabrol, Jayne Antony, Lesley Ades, Alistair T. Pagnamenta, Adam Jackson, Sofia Douzgou, Genomics England Research Consortium, J. C. Ambrose, P. Arumugam, M. Bleda, F. Boardman-Pretty, C. R. Boustred, H. Brittain, M. J. Caulfield, G. C. Chan, T. Fowler, A. Giess, A. Hamblin, S. Henderson, T. J. P. Hubbard, R. Jackson, L. J. Jones, D. Kasperaviciute, M. Kayikci, A. Kousathanas, L. Lahnstein, S. E. A. Leigh, I. U. Leong, F. J. Lopez, F. Maleady-Crowe, L. Moutsianas, M. Mueller, N. Murugaesu, A. C. Need, P. O’Donovan, C. A. Odhams, C. Patch, D. Perez-Gil, M. B. Pereira, J. Pullinger, T. Rahim, A. Rendon, T. Rogers, K. Savage, K. Sawant, R. H. Scott, A. Siddiq, A. Sieghart, S. C. Smith, A. Sosinsky, A. Stuckey, M. Tanguy, E. R. A. Thomas, S. R. Thompson, A. Tucci, E. Walsh, M. J. Welland, E. Williams, K. Witkowska, S. M. Wood, Christian Beetz, Vasiliki Karageorgou, Barbara Vona, Aboulfazl Rad, Jamshaid Mahmood Baig, Tipu Sultan, Javeria Raza Alvi, Shazia Maqbool, Fatima Rahman, Mehran Beiraghi Toosi, Farah Ashrafzadeh, Shima Imannezhad, Ehsan Ghayoor Karimiani, Yasra Sarwar, Sheraz Khan, Muhammad Jameel, Angelika A. Noegel, Birgit Budde, Janine Altmüller, Susanne Motameny, Wolfgang Höhne, Henry Houlden, Peter Nürnberg, Bernd Wollnik, Laurent Villard, Fowzan Sami Alkuraya, Matthew Osmond, Muhammad Sajid Hussain, Gökhan Yigit

**Affiliations:** 1 Cologne Center for Genomics (CCG), University of Cologne and University Hospital Cologne, Cologne, Germany; 2 Institute of Biochemistry I, Medical Faculty, University of Cologne, Cologne, Germany; 3 Human Molecular Genetics Laboratory, Health Biotechnology Division, National Institute for Biotechnology and Genetic Engineering (NIBGE) College, PIEAS, Faisalabad, Pakistan; 4 Department of Neuromuscular Disorders, UCL Institute of Neurology, London, UK; 5 Department of Medical Genetics, Ankara Bilkent City Hospital, Ankara, Turkey; 6 Aix Marseille Univ, INSERM, MMG, Marseille, France; 7 Assistance Publique–Hôpitaux de Marseille, Hôpital La Timone Enfants, Département de Génétique Médicale, Marseille, France; 8 Genetics of Learning Disability Service, Hunter Genetics, Waratah, NSW Australia; 9 Manchester Centre for Genomic Medicine, St Mary’s Hospital, Manchester University NHS Foundation Trust, Health Innovation Manchester, Manchester, UK; 10 Division of Evolution and Genomic Sciences, School of Biological Sciences, Faculty of Biology, Medicine and Health, University of Manchester, Manchester, UK; 11 Department of Pediatrics, College of Medicine, Imam Abdulrahman Bin Faisal University, Dammam, Saudi Arabia; 12 Institute of Human Genetics, University Medical Center Göttingen, Göttingen, Germany; 13 Paediatric Genetic and Metabolic Service, Tawam Hospital, Al Ain, United Arab Emirates; 14 Department of Biological and Biomedical Sciences, Aga Khan University, Karachi, Pakistan; 15 Pakistan Science Foundation (PSF), Islamabad, Pakistan; 16 Children’s Hospital of Eastern Ontario Research Institute, University of Ottawa, Ottawa, Canada; 17 University Institute of Biochemistry and Biotechnology (UIBB), PMAS-Arid Agriculture University, Rawalpindi, Pakistan; 18 Neurochemicalbiology and Genetics Laboratory (NGL), Department of Physiology, Faculty of Life Sciences, Government College University, Faisalabad, Pakistan; 19 Centre for Biotechnology and Microbiology, University of Swat, Swat, Pakistan; 20 Assistance Publique–Hôpitaux de Marseille, APHM, Hôpital Timone Enfants, Service de Neurologie Pédiatrique, Marseille, France; 21 T.Y. Nelson Department of Neurology and Neurosurgery, The Children’s Hospital at Westmead, Sydney, Australia; 22 Specialty of Child and Adolescent Health and Discipline of Genomic Medicine, The Children’s Hospital at Westmead Clinical School, University of Sydney, Sydney, Australia; 23 Department of Clinical Genetics, The Children’s Hospital at Westmead, Sydney, Australia; 24 National Institute for Health Research Oxford Biomedical Research Centre, Wellcome Centre for Human Genetics, University of Oxford, Oxford, UK; 25 CENTOGENE GmbH, Rostock, Germany; 26 Department of Otolaryngology, Head and Neck Surgery, Tübingen Hearing Research Centre (THRC), Eberhard Karls University Tübingen, Tübingen, Germany; 27 Department of Bioinformatics & Biotechnology, Faculty of Basic and Applied Sciences, International Islamic University, Islamabad, Pakistan; 28 Department of Pediatric Neurology, Children’s Hospital and Institute of Child Health, Lahore, Pakistan; 29 Development and Behavioural Pediatrics Department, Institute of Child Health and The Children Hospital, Lahore, Pakistan; 30 Pediatric Neurology Department, Ghaem Hospital, Mashhad University of Medical Sciences, Mashhad, Iran; 31 Molecular and Clinical Sciences Institute, St. George’s, University of London, Cranmer Terrace, London, UK; 32 Innovative Medical Research Center, Mashhad Branch, Islamic Azad University, Mashhad, Iran; 33 Center for Molecular Medicine Cologne (CMMC), University of Cologne, Faculty of Medicine, University Hospital Cologne, Cologne, Germany; 34 Cluster of Excellence “Multiscale Bioimaging: from Molecular Machines to Networks of Excitable Cells” (MBExC), University of Göttingen, Göttingen, Germany; 35 Department of Translational Genomics, Center for Genomic Medicine, King Faisal Specialist Hospital and Research Center, Riyadh, Saudi Arabia; 36 Department of Anatomy and Cell Biology, College of Medicine, Alfaisal University, Riyadh, Saudi Arabia; 37 Genomics England, London, UK; 38 William Harvey Research Institute, Queen Mary University of London, London, UK

## Abstract

**Purpose:**

We aimed to define a novel autosomal recessive neurodevelopmental disorder, characterize its clinical features, and identify the underlying genetic cause for this condition.

**Methods:**

We performed a detailed clinical characterization of 19 individuals from nine unrelated, consanguineous families with a neurodevelopmental disorder. We used genome/exome sequencing approaches, linkage and cosegregation analyses to identify disease-causing variants, and we performed three-dimensional molecular in silico analysis to predict causality of variants where applicable.

**Results:**

In all affected individuals who presented with a neurodevelopmental syndrome with progressive microcephaly, seizures, and intellectual disability we identified biallelic disease-causing variants in Protocadherin-gamma-C4 (*PCDHGC4*). Five variants were predicted to induce premature protein truncation leading to a loss of PCDHGC4 function. The three detected missense variants were located in extracellular cadherin (EC) domains EC5 and EC6 of PCDHGC4, and in silico analysis of the affected residues showed that two of these substitutions were predicted to influence the Ca^2+^-binding affinity, which is essential for multimerization of the protein, whereas the third missense variant directly influenced the *cis*-dimerization interface of PCDHGC4.

**Conclusion:**

We show that biallelic variants in *PCDHGC4* are causing a novel autosomal recessive neurodevelopmental disorder and link PCDHGC4 as a member of the clustered PCDH family to a Mendelian disorder in humans.

## INTRODUCTION

Protocadherins (PCDHs) comprise a large family of over 80 cell surface receptors that are mainly expressed during the development of the vertebrate nervous system and play a crucial role in the discrimination between self and nonself cell surface identities in the course of establishment and generation of neuronal circuits [1, 2]. Based on their genomic organization, human PCDHs can be divided into two families which are either encoded by genes distributed across the genome (nonclustered PCDHs) or genes clustered in a 1-Mb region on human chromosome 5 [3]. Clustered PCDHs (cPCDH) are encoded by a total of 53 genes arranged in three subclusters (*PCDHA*, *PCDHB*, and *PCDHG*) within this region [4–6]. All cPCDHs have a similar structure. They are type I transmembrane proteins containing six extracellular cadherin (EC) domains, a transmembrane region, and, in case of α- and γ-PCDH, an intracellular domain (ICD) [1]. In the *PCDHA* and *PCDHG* subclusters, multiple “variable” exons, that encode for the entire extracellular region, the transmembrane domain and a variable part of the intracellular region, are tandemly arranged upstream of three “constant” exons, which are shared within a subcluster and code for a common C-terminal intracellular domain [4, 7]. cPCDHs are widely expressed in the developing and mature nervous system including the spinal cord, cerebellum, and hippocampus [8–11]. They have been shown to form homophilic *cis*- and *trans*-interactions inducing the formation of multimeric protein complexes [12–14]. Neurons have been suggested to create a unique “barcode” by the expression of different combinations of these proteins that results in the generation of neuron-specific sets of *cis*-dimers and allows self–nonself discrimination based on the formation of *trans*-homophilic interactions [2, 15]. Recent functional studies have linked numerous cPCDHs to critical neuronal processes such as regulation of neuronal survival, axon outgrowth and targeting, dendrite arbor complexity, self-avoidance of sister axon and dendrite branches, and synaptogenesis [8, 16–18]. Whereas knockout mice of the *α-Pcdh* cluster are viable and fertile and show only abnormal axonal projections of serotonergic and olfactory sensory neurons [16, 19], disruption of the *γ-Pcdh* locus leads to neonatal lethality [8, 20, 21]. Recent studies revealed that *Pcdhgc3*, *Pcdhgc4*, and *Pcdhgc5* are crucial for the observed lethality [22, 23].

Hitherto, rare variants in nonclustered PCDH have been identified in individuals with different neurodevelopmental disorders. Rare biallelic variants in *PCDH12* (OMIM 605622) and *PCDH15* (OMIM 605514) have been reported in patients with diencephalic–mesencephalic junction dysplasia syndrome 1 (DMJDS1; OMIM 251280), Usher syndrome type 1F (USH1F, OMIM 602083), and nonsyndromic hearing loss (DFNB23; OMIM 609533), respectively [24, 25]. Furthermore, more than 100 disease-causing variants have been described in *PCDH19* (MIM 300460) in developmental and epileptic encephalopathy 9 (DEE9, OMIM 300088), making it one of the clinically relevant genes in epilepsy [26]. So far, no disease-causing variant has yet been identified in any of the cPCDHs to be causative for a Mendelian disorder in humans, despite their important role during neurodevelopment and in neural circuit assembly. In this study, we report the identification of biallelic disease-causing variants in Protocadherin-gamma-C4 (*PCDHGC4*) in 19 individuals from nine unrelated families. Affected individuals presented with progressive microcephaly, global developmental delay, intellectual disability, seizures, joint anomalies, and additional dysmorphic features. These findings establish biallelic *PCDHGC4* variants as genetic cause for a novel neurodevelopmental disorder in humans, and elucidate the associated phenotype.

## MATERIALS AND METHODS

### Subjects

Individuals who participated in this study were clinically characterized in several clinics across the world (see Supplemental Information), and we used the GeneMatcher tool [27] to connect centers in which genetic analyses were performed. All individuals reported herein are born to consanguineous families of different geographic origin, and respective families were not related to each other. Subjects or their legal representatives gave written informed consent for the molecular analyses, publication of the results and clinical information, including photographs. All studies were performed in accordance with the Declaration of Helsinki protocols and were reviewed and approved by the local institutional ethics board. DNA from participating family members was extracted from peripheral blood lymphocytes by standard extraction procedures.

### Genome/exome sequencing and linkage analysis

Genome and exome sequencing was performed on patient/parent trios (family 8), single (family 9), or multiple affected family members (families 1–7). Details on sequencing and variant screening as well as genome-wide linkage analysis (family 1) are provided as Supplementary Information.

### Variant verification and Sanger sequencing

Verification of identified nonsense and missense variants was performed using standard methods for polymerase chain reaction (PCR) amplification and Sanger sequencing. Primer sequences are available on request. The coding sequence of *PCDHGC4* (NM_018928.2) was analyzed and variants were confirmed by a second PCR on an independent DNA sample and analyzed for cosegregation within the respective families.

### Prediction programs

In silico prediction of the mutational effect for all missense variants was performed using Combined Annotation Dependent Depletion (CADD; https://cadd.gs.washington.edu), MutationTaster (www.mutationtaster.org), PolyPhen-2 (http://genetics.bwh.harvard.edu/pph2), and SIFT (https://sift.bii.a-star.edu.sg). Variants with potential effects on splicing were characterized using ESEfinder and RESCUE ESE (see Supplemental Information).

### Structural analysis of mouse Pcdhgb7 and in silico analysis of the mutational effect

Crystal structure of the Ca^2+^-bound form of mouse Pcdhgb7 was obtained from the Protein Data Bank (www.wwpdb.org; PDB ID 5v5x). Structural analysis, data visualization, and figure preparation were carried out with the program PyMOL 2.3 (www.pymol.org; Schrödinger, LLC) and WebLab viewerPro (Molecular Simulations Inc.).

## RESULTS

### Clinical presentation of individuals with a novel neurodevelopmental phenotype

**Fig. 1:**
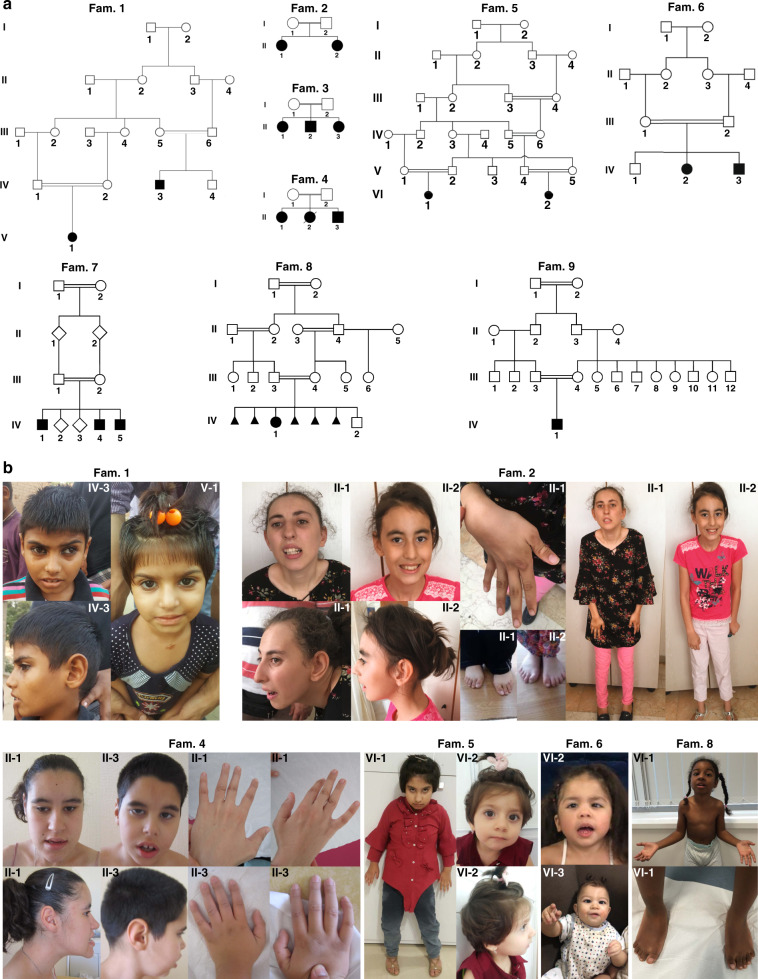
Pedigrees and clinical characteristics of individuals harboring biallelic disease-causing variants in *PCDHGC4*. (**a**) Pedigrees of nine unrelated families with disease-causing variants in *PCDHGC4*. All affected siblings (solid symbols) in each family carry homozygous disease-causing variants in *PCDHGC4* while unaffected  parents are heterozygous for identified *PCDHGC4* variants (white symbols). (**b**) Upper panel: facial features of subjects IV-3 and V-1 from family 1 (left), clinical characteristics of subjects II-1 and II-2 from family 2 showing kyphoscoliosis, clinodactyly and hallux valgus (subject II-1), and kyphosis and hypoplasia of the toes (subject II-2). Lower panel (from left to right): facial features and hand anomalies observed in subjects II-1 (22 years) and II-3 (14 years) from family 4, clinical characteristics of subjects VI-1 and VI-2 from family 5, and subjects IV-2 and IV-3 from family 6, and facial features and feet anomalies observed subject VI-1 from family 8.

In a national and international collaboration, we recruited 19 individuals from nine unrelated families with a clinical diagnosis of a neurodevelopmental disorder. Clinical findings on all affected individuals are summarized in Table 1, with pedigrees and clinical photographs shown in Fig. 1. Comprehensive clinical information on families (1–4, 7, 8) is provided as Supplemental Information. For five individuals (families 5, 6, and 9), no extensive clinical descriptions are available.

**Table 1:** Summary of genetic data and clinical features of affected individuals.

Pedigree ID	Family 1		Family 2		Family 3			Family 4		
	IV-3	V-1	II-1	II-2	II-1	II-2	II-3	II-1	II-2	II-3
Age^a^ (years)	10	8	20	11	30	27	24	22	1 6/12^b^	14
Gender	Male	Female	Female	Female	Female	Male	Female	Female	Female	Male
Geographic origin	Pakistan	Pakistan	Turkey	Turkey	Iraq	Iraq	Iraq	Morocco	Morocco	Morocco
Parental consanguinity	+	+	+	+	+	+	+	+	+	+
*PCDHGC4* variant	c.1449C>G; p.(Asp483Glu)	c.1449C>G; p.(Asp483Glu)	c.118C>T; p.(Gln40*)	c.118C>T; p.(Gln40*)	c.1463C>T; p.(Ala488Val)	c.1463C>T; p.(Ala488Val)	c.1463C>T; p.(Ala488Val)	c.1817T>G; p.(Val606Gly)	c.1817T>G; p.(Val606Gly)	c.1817T>G; p.(Val606Gly)
**Birth**										
Gestation (weeks)	38	38	40	39	40	40	40	41	NR	41
Weight (g)	3,000 (−0.4 SD)	2,500 (−1.3 SD)	3,850 (0.8 SD)	3,500 (0.5 SD)	Within normal limits	Within normal limits	Within normal limits	3,020 (−1 SD)	NR	3,300 (−1 SD)
Height (cm)	NR	NR	45 (−2.4 SD)	50 (0.2 SD)	Within normal limits	Within normal limits	Within normal limits	45 (−3 SD)	NR	48 (−2 SD)
Head circumference (cm)	NR	NR	34 (−0.5 SD)	35 (0.6 SD)	NR	NR	NR	34 (−1 SD)	NR	34 (−1 SD)
**Clinical characteristics**										
Facial dysmorphism	–	–	Sloping forehead, triangular asymmetric face, high nasal bridge, high narrow palate	High narrow palate, high nasal bridge	–	–	–	Long face, round ears with attached lobes, thick lips with everted inferior lips, mild prognathism	NR	Long face, round ears with attached lobes, thick lips with everted inferior lips, mild prognathism
Acral anomalies	–	–	Clinodactyly, hallux valgus	Hypoplasia of toes	Broad thumbs, swan neck deformity	Broad thumbs, swan neck deformity	Swan neck deformity	Bilateral ulnar clubhand, valgus deformities	Bilateral ulnar clubhand	Bilateral ulnar clubhand, flexion contracture of legs and clubfoots
Seizures	–	+	+; at 2, 4 and 7 years of age	+; at 1.5 and 6.5 years of age	–	–	+; (singular) febrile seizure	+; generalized seizures starting at 5 years	+; status epilepticus at 18 months	+; generalized seizures starting at 18 months
Brain MRI anomalies	Thin cerebral cortex	NR	Cerebral atrophy	–	NR	NR	NR	Mild cortical atrophy	NR	Cortical atrophy
Hearing impairment	–	–	–	–	–	–	–	–	NR	–
Ocular anomalies	Strabismus	–	–	–	–	–	–	–	NR	–
Additional findings	Gait abnormalities	Gait abnormalities, recurrent infections	Kyphoscoliosis	Kyphosis	Hypotonic at birth, unsteady gait, hyperextensible joints	Hypotonic at birth, unsteady gait, hyperextensible joints, white hypopigmented patch	Hypotonic at birth, unsteady gait, hyperextensible joints;	Hypotonic at birth, lumbar scoliosis	Hypotonic at birth	Arthrogryposis
Intellectual disability	Moderate	Severe	Moderate	Mild	Severe	Severe	Severe	Moderate	NR	Severe
Development	Speech impairment, started walking at 4 years of age	Aggressive behavior, poor self-care	Hyperactivity	Hyperactivity	Global developmental delay, able to speak 2–3 words, able to toilet and dress with assistance; started walking between 7 and 10 years	Global developmental delay, nonverbal, able to toilet and dress with assistance; started walking between 7 and 10 years	Global developmental delay, able to toilet and dress with assistance; started walking between 7 and 10 years	Global developmental delay, walked at the age of 2 years; able to speak with a lot of words and to copy a text, able to toilet and to dress	Global developmental delay, no language development at 18 months, deceased at 18 months	Global developmental delay, nonverbal, uses signs and gestures to communicate; walked at 10 years of age, able to dress with assistance
**Current measurements**										
Head circumference (cm)	45 (−4.7 SD)	45 (−4.5 SD)	53	49	51 (−2.5 SD)	51 (−3.7 SD)	49 (−3.9 SD)	52 (−2 SD)	NR	51 (−2 SD)
Height (cm)	127 (−2 SD)	113 (−2.5 SD)	153	149	150 (−2.1 SD)	164 (−1.8 SD)	150 (−2.1 SD)	152 (−2 SD)	NR	138 (−2 SD)
Weight (kg)	NR	NR	NR	NR	39	38.5	32.4	48	NR	38

Pedigree ID	Family 5		Family 6		Family 7			Family 8	Family 9
	VI-1	VI-2	IV-1	IV-3	III-1	III-4	III-5	IV-1	IV-1
Age^a^ (years)	8 5/12	1 6/12	3	1	17	6	2	11	1 10/12
Gender	Female	Female	Female	Male	Male	Male	Male	Female	Male
Geographic origin	Iran	Iran	Saudi Arabia	Saudi Arabia	Lebanon	Lebanon	Lebanon	Sudan	Jordan
Parental consanguinity	+	+	+	+	+	+	+	+	+
*PCDHGC4* variant	c.324del; p.Phe108Leufs*14	c.324del; p.Phe108Leufs*14	c.1463C>T; p.(Ala488Val)	c.1463C>T; p.(Ala488Val)	c.1243C>T; p.(Arg415*)	c.1243C>T; p.(Arg415*)	c.1243C>T; p.(Arg415*)	c.1724dup; p.(Leu575Phefs*63)	c.2443-1G>A^c^
**Birth**									
Gestation (weeks)	40	41	At term	39	42	39	38	40	At term
Weight (g)	2,460 (−3 SD)	3,700 (0.2 SD)	3,600 (0.4 SD)	3,900 (1.1 SD)	3,645 (−0.7 SD)	3,325 (−0.1 SD)	3,280 (0.3 SD)	2,330 (−2.5 SD)	NA
Height (cm)	45 (−3 SD)	53 (0.8 SD)	NR	51 (0.3 SD)	52 (−0.4 SD)	51 (0.3 SD)	49 (−0.1 SD)	NR	NA
Head circumference (cm)	32 (−3 SD)	35.5 (0.2 SD)	NR	37 (1.7 SD)	34 (−1.5 SD)	35 (0.3 SD)	35 (0.3 SD)	32.4 (−1.7 SD)	NA
**Clinical characteristics**									
Facial dysmorphism	Mild metopic craniosynostosis, everted inferior lips	Mild metopic craniosynostosis	Epicanthic fold, high nasal bridge, low set ears, overlapping teeth	Epicanthic fold, high nasal bridge, low set ears, overlapping teeth	Doliocephaly, mild synophrys, upslanting palpebral fissures, long eyelashes, high nasal bridge, hooked nose, overhanging columella, short philtrum, full lips, high narrow palate, mild malar hypoplasia	Mild synophrys, long eyelashes, high nasal bridge, broad nasal bridge and nasal tip, slightly overhanging columella, short philtrum, full lips	Mild synophrys, long eyelashes, high nasal bridge, short philtrum, full lips	Microcephaly, receding forehead	Mildly dysmorphic chin, low nasal bridge, small, upturned nose, long philtrum, metopic craniosynostosis
Acral anomalies	Broad thumbs	–	–	–	Persistent fetal fingertip pads, mild 2nd and 5th clinodactyly	Mild 2nd and 5th finger clinodactyly, small broad feet	–	–	Bilateral thumb-in-palm deformity, rigid equinovarus deformity (right foot), fixed rocker bottom deformity with severe calcaneovalgus (left foot)
Seizures	+; tonic seizures starting at 18 months, last seizure at 8 3/12 years	+; neonatal, tonic seizures	–	–	–	–	–	+; febrile seizures	–
Brain MRI anomalies	Mild extra axial widening (at 4 6/12 years of age)	Mild extra axial widening	Left-sided hypodensities within the temporal lobe and left cerebral hemisphere as results of previous microhemorrhages; CSF space enlargement	Acute ischemic insult within the left thalamus and left parietal region	Left temporal fossa arachnoid cyst, mild ventriculomegaly and thinning of body and genu of corpus callosum	Mild ventriculomegaly and thinning of anterior body of corpus callosum	NR	Frontal horns mildly prominent with mild atrophy (at 7 years)	NR
Hearing impairment	–	–	–	–	–	–	–	–	–
Ocular anomalies	Microphthalmus, ptosis of the right eye	Exotropia, mild oculomotor apraxia	Esotropia	Esotropia	Oculomotor apraxia	Oculomotor apraxia	Oculomotor apraxia	Oculomotor apraxia	Oculomotor apraxia, restricted ocular motility, bilateral ptosis, axial myopia of the right eye, myopic astigmatism of the left eye
Additional findings	DDH	DDH	Spasticity and truncal hypotonia, bilateral DDH	Spasticity and truncal hypotonia, hip dislocation (left)	Neonatal hypertonia, 15^o^ contractures at elbows, hypoplastic calves, mild genu valgum, brisk reflexes	Neonatal hypertonia, exaggerated startle response	Neonatal hypertonia, exaggerated startle response	Arthrogryposis multiplex congenita (mainly elbows and ankles), slow weight gain, vitamin D deficiency	Arthrogryposis multiplex congenital (shoulders elbows, hips, and knees)
Intellectual disability	Severe	Moderate	–	–	Moderate	Moderate	Mild	Moderate	Mild
Development	Global developmental delay, started walking at the age of 4 years, only babbling	Global developmental delay, can stand with support	Global developmental delay, severe receptive and expressive language delay	Global developmental delay	Severe speech delay and articulation problems, moderate intellectual disability	Absent speech, global developmental delay	Severe speech delay, global developmental delay	Walking at age 5 years 4 months, speech delay, single words at age 10 years, understands simple conversations and commands in English and Arabic	Global developmental delay, no smiling, reduced social interaction
**Current measurements**									
Head circumference (cm)	48 (−3.6 SD)	43 (−2.4 SD)	44 (−2.5 SD)	41 (−2.5 SD)	56.5 (0.3 SD)	51 (−0.9 SD)	47 (−1.2 SD)	46.5 (−3.72 SD)	NA
Height (cm)	127 (−0.5 SD)	67 (−4.7 SD)	81 (−2.5 SD)	63.5 (−2.1 SD)	155.5 (−2.9 SD)	126 (+0.4 SD)	95 (−1.3 SD)	104 (−2.87 SD)	NA
Weight (kg)	35 (0.8 SD)	9 (−1.0 SD)	28 (−3.4 SD)	7.3 (−0.6 SD)	72	21	13	15.1 (−2.90 SD)	NA

*CSF* cerebrospinal fluid, *DDH* developmental dysplasia of the hip, *IUGR* intrauterine growth retardation, *MCKD* medullary cystic kidney disease, *MRI* magnetic resonance image, *NR* not recorded, *OFC* occipital–frontal circumference, *SD* population-based standard deviations.

^a^Age at the time of latest examination.

^b^Deceased.

^c^Position referring to *PCDHGC4* (NM_018928.2, ENST00000306593.1).

**Fig. 2:**
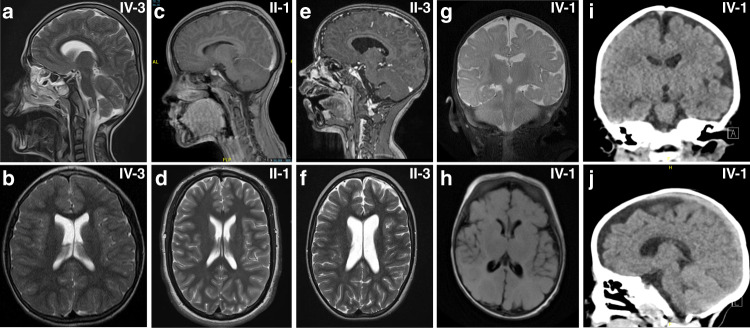
Neuroradiologic features of affected individuals. Sagittal (**a**) and axial (**b**) T2-weighted images of subject IV-3 from family 1 at the age of 10 revealed no structural brain anomalies but showed microcephaly and thin cerebral cortex. (**c**) Sagittal T1 section after gadolinium injection of subject II-1 (family 4) at 10 years of age and (**d**) axial T2-weighted images at 16 years revealed no brain-specific abnormality except for a discreet prominent aspect of the lateral ventricles. (**e**) Sagittal T1 section after gadolinium injection and (**f**) axial T2-weighted images of subject II-3 (family 4) at 7 years of age revealed a prominent aspect of the lateral ventricles, of the 3rd and, to a milder degree, of the 4th ventricle. (**g**) Coronal T2-weighted and (**h**) axial T2-Flair images of subject IV-2 (family 6) at 3 months of age showing normal signal intensity, age-appropriate myelination process and slightly enlarged cerebrospinal fluid (CSF). Coronal (**i**) and sagittal (**j**) computed tomography (CT) images of the same subject at the age of 3 years revealing left-sided subcortical hypodensity within left temporal lobe and confirming prominent CSF space.

Common features in our patient cohort were developmental delay (DD)/intellectual disability (ID) (18/19), microcephaly (12/19), seizures (10/19), hypotonia (10/19), and skeletal/joint anomalies (10/19). Occipital–frontal circumferences (OFCs) at birth ranged from 1.7 SD (individual IV-3, family 6) to −3 SD (individual VI-2, family 5), and we observed microcephaly at birth (OFC ≤ −2 SD) in 2/19 patients. However, at follow up examinations, 12/19 individuals showed progressive mild to severe microcephaly with values from −2 SD to −5.5 SD. Neuroimaging was available for 12 individuals. Brain magnetic resonance image/computed tomography (MRI/CT) abnormalities (11/12 patients) were rather nonspecific (Fig. 2, Table 1). Microcephaly, thin cerebral cortex, mild ventriculomegaly, and cortical atrophy were the commonest features. Seizure types ranged from singular febrile seizures (family 3, subject II-3), recurring events (family 2, subjects II-1 and II-2) to generalized tonic, clonic–focal to multifocal seizures (families 4 and 5; Table 1). Electroencephalogram (EEG) data was available for four subjects (family 2, subjects II-1 and II-2; family 5, subject VI-2; family 7, subjects III-1) and showed no abnormalities. ID and DD were present in all our patients, and we observed motor and speech developmental delay as well as mild to severe cognitive impairment. Three individuals presented with kyphosis and/or scoliosis, hyperextensible joints were observed in three individuals and contractures as wells as arthrogryposis were present in four individuals (Fig. 1b, Table 1). Dysmorphic facial features were rather nonspecific and did not reveal a common, recognizable facial presentation within our patient cohort (Fig. 1b, Table 1).

### Identification of biallelic truncating and missense *PCDHGC4* variants

**Fig. 3:**
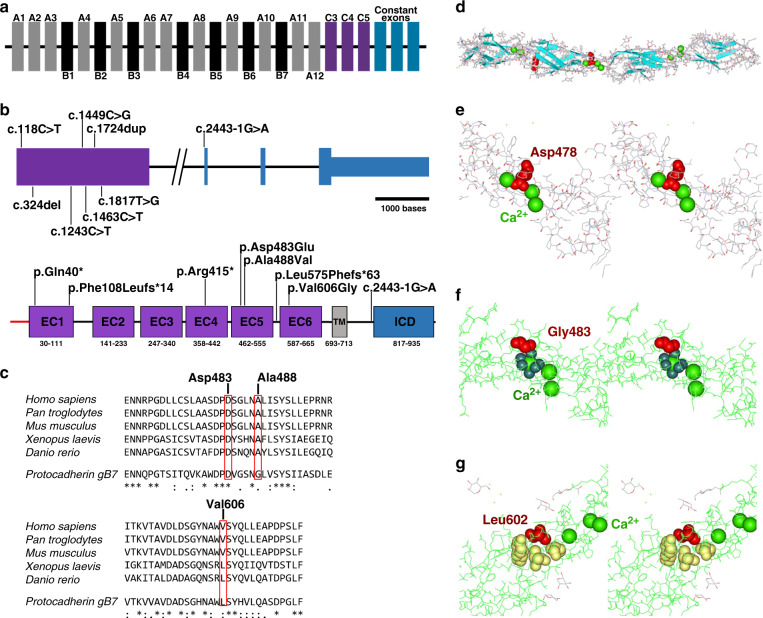
Molecular characterization and in silico analysis of identified disease-causing variants in *PCDHGC4*. (**a**) Schematic representation of the human *γ-PCDH* cluster. Variable exons of the *γ-PCDH* A and B subfamilies are shown in gray and black, respectively. Variable exons of the *γ-PCDH* C subfamily are shown in purple, *γ-PCDH* constant exons in blue. (**b**) Schematic representation of the genomic (upper panel) and protein structure (lower panel) of PCDHGC4, and localization of the identified disease-causing variants. Introns are shown by black horizontal line, coding exons by purple and blue bars, noncoding regions of exons by small blue bar (upper panel). Scale bar is referring solely to exons. Protein structure of PCDHGC4 with six extracellular cadherin (EC) repeats (purple), the transmembrane region (gray), and the intracellular domain (ICD, blue). (**c**) Amino acid sequence alignment of PCDHGC4 across different species including mouse Pcdhgb7 (lower line, all panels) for residues p.Asp483 and p.Ala488 (upper panel) and p.Val606 (lower panel) that are altered in the affected subjects. Protein sequences were prepared from UniProtKB and alignment was performed using Clustal Omega. Position of the altered residues in human are indicated (top numbers). (**d**) Three-dimensional structure of the EC3 to EC6 domains of Pcdhgb7. Structural information was obtained from the Protein Data Bank (PDB) and is available under the accession number 5v5x. Pcdhgb7 is shown in ribbon representation. β-strands are shown as arrows (blue), a short helical part in red, calcium ions in sphere representation (green), and aspartate at position 478 within the Ca^2+^-binding DXD motif in space filling representation (red). (**e**-**g**) Close up crossed eyes stereo views of p.Asp478 in Pcdhgb7 corresponding to p.Asp483 in PCDHGC4 (**e**), p.Gly483 (corresponding to p.Ala488 in PCDHGC4) (**f**), and p.Leu602 (corresponding to p.Val606 in PCDHGC4) (**g**). Affected amino acid residues are labeled in red, calcium ions are shown in sphere representation (light green), oxygen ligands of the adjacent calcium ion in space filling representation (**f**, dark green), surrounding hydrophobic residues p.Pro558, p.Tyr604, and p.Val644 of p.Leu602 in space filling representation in yellow (**g**).

We performed linkage analysis (family 1) and/or genome/exome sequencing in probands and proband/parent trios. Based on parental consanguinity, autosomal recessive inheritance was considered likely, and we prioritized homozygous, rare exonic, and splice site variants (see Supplemental Information). We identified three different missense variants and five protein truncating variants in the Protocadherin-gamma family member *PCDHGC4* (OMIM 606305; NM_018928.2) in all affected individuals (Fig. 3a, b, S1, Table 2). All variants fully cosegregated with the phenotype in the respective families and are absent or very rare in the general human population with minor allele frequencies (MAFs) ranging from 0 to 4*10^−6^, in line with an autosomal recessive pattern of inheritance (Table 2). We identified four homozygous loss-of-function variants in *PCDHGC4*, c.118C>T (p.[Gln40*]), c.324del (p.[Phe108Leufs*14]), c.1243C>T (p.[Arg415*]), c.1724dup (p.[Leu575Phefs*63]), that were predicted to lead to an early stop and premature protein truncation, and were absent from the gnomAD database (Fig. 3b, Table 2). In family 9, we found the homozygous variant c.2443-1G>A at the acceptor splice site of intron 1, and by employing an exon‐trapping approach we could show that this variant leads to a loss of the acceptor splice‐site recognition resulting in severe splicing defects such as whole‐exon skipping or usage of a cryptic exonic acceptor splice site, which both are predicted to induce a frameshift and premature protein truncation (Fig. 3b, S3). Within the family of γ-PCDHs, PCDHGC4 is the only member that is not only highly conserved across species, but also under strict mutational constraint [23]. Truncating variants in *PCDHGC4* are rarely observed in healthy control individuals. For the canonical transcript of *PCDHGC4* (ENST00000306593.1, NM_018928.2) only 12 alleles with nonsense variants, all in heterozygous state, were reported in the gnomAD database in contrast to 29.6 that were expected to be observed in the >240,000 alleles (probability of loss of function intolerance [pLI] = 0.98). Further, biallelic copy-number variants (CNVs) encompassing *PCDGHC4* have not been reported so far in the DECIPHER database, the Database of Genomic Variants (DGV), and the structural variant (SV) data set of gnomAD with only two (DGV) and six (gnomAD) heterozygous alterations enlisted in these data sets that affect *PCDHGC4*. Interestingly, genetic disruption of the entire *γ-Pcdh* cluster as well as singular knockout of *Pcdhgc4* in mice also cause a severe neurodevelopmental phenotype, both resulting in neurodegeneration in late embryonic stages and leading to early neonatal lethality [8, 20–23].

**Table 2:** In silico prediction and population allele frequencies of *PCDHGC4* (NM_018928.2; ENST00000306593.1) variants identified in this study.

Family	Genomic location (GRCh37/hg19)	HGVS cDNA	HGVS protein	Allele frequency (gnomAD database^a^)	Prediction scores			
					SIFT^b^	PolyPhen-2^c^	MutationTaster^d^	CADD
**1**	chr5:140866189	c.1449C>G	p.(Asp483Glu)	0	D 0.000	PD 1.00	Polymorphism 0.932	24.1
**2**	chr5:140864858	c.118C>T	p.(Gln40*)	0	NA	NA	DC 1.000	NA
**3;6**	chr5:140866203	c.1463C>T	p.(Ala488Val)	0.000004	D 0.003	PD 0.971	Polymorphism 0.528	25.2
**4**	chr5:140866557	c.1817T>G	p.(Val606Gly)	0	D 0.000	PD 0.968	DC 0.999	26.9
**5**	chr5:140865064	c.324del	p.(Phe108Leufs*14)	0	NA	NA	DC 1.000	NA
**7**	chr5:140865983	c.1243C>T	p.(Arg415*)	0	NA	NA	DC 1.000	NA
**8**	chr5:140866464	c.1724dup	p.(Leu575Phefs*63)	0	NA	NA	DC 1.000	NA
**9**	chr5:140874373	c.2443-1G>A	NA	0	NA	NA	DC 1.000	NA

*cDNA* complementary DNA, *D* deleterious, *DC* disease causing, *NA* not applicable, *PD* probably damaging.

^a^Accessed in January 2021.

^b^Score 1–0.

^c^HumVar prediction, score 0–1.

^d^Score 0–1.

Furthermore, we identified three different homozygous missense variants, c.1449C>G (p.[Asp483Glu]), c.1463C>T (p.[Ala488Val]), and c.1817T>G (p.[Val606Gly]), in *PCDHGC4* in affected individuals of four additional consanguineous families (Fig. 3, Table 2). In silico prediction of the pathogenic effect of these missense variants by different prediction tools leads to the classification as damaging (SIFT), probably damaging (PolyPhen-2), and a Combined Annotation Dependent Depletion (CADD) score of 24.1 to 26.9, indicating deleteriousness of these variants (Table 2). Two missense variants, p.(Asp483Glu) and p.(Ala488Val), were classified as polymorphisms by a single in silico prediction tool, MutationTaster. In two families, families 3 and 6 from Iraq and Saudi Arabia, respectively, we identified the identical missense variant, c.1463C>T (p.[Ala488Val]), in *PCDHGC4*. In affected individuals of both families, this variant was within a shared haplotype of approximately 309 kb between chr5:140,750,044 and chr5:141,059,868 suggesting a founder nature of the variant. On protein level, the three missense variants are located in the extracellular domain of PCDHGC4 within the fifth (p.[Asp483Glu) and p.(Ala488Val]) or sixth (p.[Val606Gly]) extracellular cadherin (EC) domain and are predicted to lead to the substitution of phylogenetically highly conserved amino acids in PCDHGC4 (Fig. 3c). EC domains are extracellular Ca^2+^-binding domains, which upon Ca^2+^ binding can mediate conformational changes influencing the rigidity of the EC domains of PCDHGC4, which enables *cis*- and *trans-*homophilic interactions [2]. Ca^2+^ binding is a crucial process for correct PCDH function. Upon binding of Ca^2+^, which is mediated by several calcium-binding motifs at the junctions of the EC repeats of PCDHs, the conformation and rigidity of these segments is controlled, allowing formation of *cis-* as well as *trans*-dimerizations [28, 29]. Whereas EC1 to EC4 contribute to the formation of head-to-tail *trans* interactions between different cells, EC5 and EC6 are involved in *cis*-dimerization processes. To gain further insights into the pathogenic effects of the missense variants, we performed an in silico analysis of the mutational effect on the protein structure using the crystal structure of mouse Pcdhgb7, a close homologue of PCDHGC4. All three missense variants were located in or directly adjacent to a Ca^2+^-binding motif. The p.(Asp483Glu) variant affects an aspartate that is part of the highly conserved DXD motif in the EC5 repeat of PCDHGC4 directly involved in calcium coordination (Fig. 3d, e). Although this variant does not change the charge of the coordinating residue, it alters the size of the residue, which is predicted to perturb the local structure and to shift the position of the coordinating carboxyl oxygens of this residue away from the optimal geometry of calcium-binding ligands. This should decrease the Ca^2+^-affinity of this motif. Interestingly, a similar substitution, p.Asp377Glu, has already been described for PCDH19, and it has been shown to impair PCDH19 function and cause early infantile epileptic encephalopathy [30]. The p.(Ala488Val) alteration is located in close proximity to the DXD motif within the fifth EC repeat (Fig. 3d, f). Structural analysis of this highly conserved residue shows that the +5 position (in relation to the DXD motif) is generally a small amino acid (glycine or alanine). Substitution of this residue with valine, as identified in our patients, introduces a large, hydrophobic amino acid, which might interfere with the adjacent Ca^2+^-binding motif, thereby impairing Ca^2+^-affinity of PCDHGC4 (Fig. 3f). Similarly, also the p.Val606 is located in proximity to a DXD motif of PCDHGC4. In contrast to the other missense variants, the p.(Val606Gly) substitution is located in the third strand of a seven-stranded β-sheet of the sixth EC domain, embedded in a hydrophobic pocket (Fig. 3g). Substitution of valine at position 606 with glycine is predicted to cause structural perturbation of this region, which potentially might impair these interactions and, as a result, directly affect the *cis*-dimerization capability of PCDHGC4. *Cis*-dimerization of γ-PCDH is not only important for *trans* homophilic interactions on the cell surface, but also essential for cell surface delivery of newly synthesized γ-PCDH itself, as demonstrated by experiments on induced mutational disruption of the *cis*-interface [31]. Currently, we can only speculate about the direct effect of these three identified missense variants on PCDHGC4 protein function, but given the fact that they are all located in the fifth or sixth EC repeat, it seems likely that they directly or indirectly influence these *cis-*dimerization processes, thereby interfering with cell surface transport of PCDHGC4-containing dimers [31].

In an additional family from Iran, we identified the homozygous missense variant c.2524G>C (p.[Gly842Ser]) in a patient presenting with facial dysmorphism, metopic craniosynostosis, ventriculomegaly, focal clonic seizures, and moderate global developmental delay (Fig. S4). This variant affects a residue within the ICD of PCDHGC4. The ICD of γ-PCDH plays an important role in the regulation of downstream signaling cascades, e.g., in the inhibition of FAK and PYK2 kinase activity, which is crucial for the promotion of dendrite arborization in cortical neurons [17]. Still, further studies are required to prove causality of this variant as well as to fully determine the specific function and involvement of this residue in intracellular, γ-PCDH-regulated signaling pathways.

## DISCUSSION

In the present study, we provide strong genetic evidence that biallelic nonsense and missense variants in *PCDHGC4* cause a distinct neurodevelopmental phenotype comprising progressive microcephaly, short stature, intellectual disability, seizures, and joint anomalies. In all 19 affected individuals from nine different families, we were able to identify homozygous disease-causing variants in *PCDHGC4* that most likely lead to a loss of function of the encoded protein.

Interestingly, we observed seizures in 10 of 19 patients. Generally, development of focal seizures is considered to be caused by a disturbance of the excitation/inhibition balance in cortical neurons. Within these neuronal circuits, GABAergic cortical inhibitory interneurons (cINs) play an important role in restraining excitation levels in the brain under normal conditions, and alterations in the number of cINs have been associated with epilepsy [32]. During embryonic development, the number of cINs is regulated by programmed cell death. Initially, excess numbers of cINs are generated from a pool of cIN progenitor cells which migrate to the developing cortex. Upon arrival, ~40% of these cells are eliminated by endogenously triggered programmed cell death [33–35]. Interestingly, except Pcdhga9, all 21 γ-Pcdhs are expressed in cINs. Expression of four isoforms, Pcdhga1, Pcdhga2, Pcdhgc4, and Pcdhgc5, increases significantly between P8 and P15, corresponding to the period in which programmed cell death of cINs takes place [36]. Recent studies showed that Pcdhgc3, Pcdhgc4, and Pcdhgc5 are crucial components in the regulation of this programmed cell death. Loss of these isoforms enhances the number of cINs undergoing apoptosis, which results in a reduced cortical density of cINs [36]. A similar function of γ-PCDHs in controlling programmed cell death has also been described for neuronal cells of the spinal cord and the retina [8, 9, 22]. Still, further molecular and cellular studies are required to determine whether disease-causing variants in *PCDHGC4* alone are sufficient to increase programmed cell death in neuronal cells and to give rise to the clinical presentation observed in our patients via this pathway. Interestingly, genetic disruption of the entire *γ-Pcdh* cluster as well as singular knockout of *Pcdhgc4* in mice both result not only in neurodegeneration in late embryonic stages but also lead to early neonatal lethality [8, 20–23]. Currently, it is unclear why disruption of *Pcdhgc4* in mice leads to neonatal lethality, whereas biallelic loss-of-function variants in human, as observed in our patients, result in a milder neurodevelopmental disorder comprising progressive microcephaly, seizures, and intellectual disability, especially when considering that both humans and mice share the same set of 22 members within the γ-PCDH cluster. But the difference between the observed phenotypes suggests that the human brain might compensate for the functional failure of PCDHGC4 resulting in a higher tolerance of loss-of-function variants in terms of lethality.

So far, to the best of our knowledge, no member of the clustered PCDH family has been shown to be involved in the pathogenesis of a congenital human disorder. In recent years, disease-causing variants in several nonclustered δ-PCDH family members have been described and closely linked to different neurodevelopmental diseases. This includes biallelic loss-of-function variants in *PCDH12* and *PCDH15*, which were identified as cause of diencephalic–mesencephalic junction dysplasia syndrome type 1 (DMJDS1), Usher syndrome type 1F, and nonsyndromic hearing loss, respectively, as well as *PCDH19*, in which over 100 different missense and nonsense variants have been reported to underlie X-linked developmental and epileptic encephalopathies 9 (DEE9) highlighting the importance of cell–cell communication via PCDH19 at the early stages of brain development [24–26, 37]. Although recent studies indicate that complete or partial epigenetic dysregulation of the clustered PCDH occurs in cells of patients with Williams–Beuren syndrome or Down syndrome, and hypermethylation of all three *PCDH* clusters is detectable in Wilms tumors, a direct link to a monogenic, congenital human disorder has not been established before [38–40]. Currently, it is unclear whether this is due to functional redundancy of the encoded PCDHs. Mice lacking the α*-* or β*-Pcdh* cluster are viable and fertile [16], whereas knockout of the whole γ-*Pcdh* cluster results in neonatal lethality [8, 20, 21]. Similar consequences were observed when only the γC3 to γC5 isoforms within this cluster were disrupted, indicating that one of these three isoforms has a critical function [22]. Very recent results based on the generation of single knockouts of γ-*Pcdh* members suggest that Pcdhgc4 is the crucial isoform within the gamma cluster, which is required for neuronal survival and responsible for neonatal lethality [23]. The unique role of *PCDHGC4* is further supported by genetic data indicating that *PCDHGC4* is the only member within the γ-*PCDH* cluster that is under strict mutational constraint [23]. We can only speculate about the molecular basis of the distinct role of PCDHGC4, especially as its overall structure is similar to other γ-PCDHs.

In conclusion, we show that biallelic truncating and missense variants in *PCDHGC4* cause a specific human phenotype characterized by neurodevelopmental delay, progressive microcephaly with mild to severe intellectual disability, global developmental delay, joint anomalies, and seizures, providing evidence that disease-causing variants in a single member of the clustered PCDH family are involved in the pathogenesis of a congenital disorder in humans.

## Supplementary Information

Supplementary Information

## Data Availability

The data that support the findings of this study are available on request from the corresponding authors. The genetic data are not publicly available due to privacy or ethical restrictions.

## References

[1] Sano K (1993). Protocadherins: a large family of cadherin-related molecules in central nervous system. EMBO J..

[2] Rubinstein R (2015). Molecular logic of neuronal self-recognition through protocadherin domain interactions. Cell..

[3] Hulpiau P, van Roy F (2009). Molecular evolution of the cadherin superfamily. Int J Biochem Cell Biol..

[4] Wu Q, Maniatis T (1999). A striking organization of a large family of human neural cadherin-like cell adhesion genes. Cell..

[5] Sugino H (2000). Genomic organization of the family of CNR cadherin genes in mice and humans. Genomics..

[6] Wu Q (2001). Comparative DNA sequence analysis of mouse and human protocadherin gene clusters.. Genome Res..

[7] Wu Q, Maniatis T (2000). Large exons encoding multiple ectodomains are a characteristic feature of protocadherin genes. Proc Natl Acad Sci USA..

[8] Wang X, Weiner JA, Levi S, Craig AM, Bradley A, Sanes JR (2002). Gamma protocadherins are required for survival of spinal interneurons. Neuron..

[9] Lefebvre JL, Kostadinov D, Chen WV, Maniatis T, Sanes JR (2012). Protocadherins mediate dendritic self-avoidance in the mammalian nervous system. Nature..

[10] Chen WV (2017). Pcdhαc2 is required for axonal tiling and assembly of serotonergic circuitries in mice. Science..

[11] Mountoufaris G (2017). Multicluster Pcdh diversity is required for mouse olfactory neural circuit assembly. Science..

[12] Murata Y, Hamada S, Morishita H, Mutoh T, Yagi T (2004). Interaction with protocadherin-gamma regulates the cell surface expression of protocadherin-alpha. J Biol Chem..

[13] Fernández-Monreal M, Kang S, Phillips GR (2009). Gamma-protocadherin homophilic interaction and intracellular trafficking is controlled by the cytoplasmic domain in neurons. Mol Cell Neurosci..

[14] Schreiner D, Weiner JA (2010). Combinatorial homophilic interaction between gamma-protocadherin multimers greatly expands the molecular diversity of cell adhesion. Proc Natl Acad Sci USA..

[15] Thu CA (2014). Single-cell identity generated by combinatorial homophilic interactions between α, β, and γ protocadherins. Cell..

[16] Hasegawa S (2008). The protocadherin-alpha family is involved in axonal coalescence of olfactory sensory neurons into glomeruli of the olfactory bulb in mouse. Mol Cell Neurosci..

[17] Garrett AM, Schreiner D, Lobas MA, Weiner JA (2012). γ-protocadherins control cortical dendrite arborization by regulating the activity of a FAK/PKC/MARCKS signaling pathway. Neuron..

[18] Molumby MJ, Keeler AB, Weiner JA (2016). Homophilic protocadherin cell-cell interactions promote dendrite complexity. Cell Rep..

[19] Katori S (2009). Protocadherin-alpha family is required for serotonergic projections to appropriately innervate target brain areas. J Neurosci..

[20] Weiner JA, Wang X, Tapia JC, Sanes JR (2005). Gamma protocadherins are required for synaptic development in the spinal cord. Proc Natl Acad Sci USA..

[21] Prasad T, Wang X, Gray PA, Weiner JA (2008). A differential developmental pattern of spinal interneuron apoptosis during synaptogenesis: insights from genetic analyses of the protocadherin-gamma gene cluster. Dev Camb Engl..

[22] Chen WV (2012). Functional significance of isoform diversification in the protocadherin gamma gene cluster. Neuron..

[23] Garrett AM (2019). CRISPR/Cas9 interrogation of the mouse Pcdhg gene cluster reveals a crucial isoform-specific role for Pcdhgc4. PLoS Genet..

[24] Guemez-Gamboa A (2018). Loss of protocadherin-12 leads to diencephalic-mesencephalic junction dysplasia syndrome. Ann Neurol..

[25] Ahmed ZM (2003). PCDH15 is expressed in the neurosensory epithelium of the eye and ear and mutant alleles are responsible for both USH1F and DFNB23. Hum Mol Genet..

[26] Dibbens LM (2008). X-linked protocadherin 19 mutations cause female-limited epilepsy and cognitive impairment. Nat Genet..

[27] Sobreira N, Schiettecatte F, Valle D, Hamosh A (2015). GeneMatcher: a matching tool for connecting investigators with an interest in the same gene. Hum Mutat..

[28] Cailliez F, Lavery R (2005). Cadherin mechanics and complexation: the importance of calcium binding. Biophys J..

[29] Sotomayor M, Schulten K (2008). The allosteric role of the Ca2+ switch in adhesion and elasticity of C-cadherin. Biophys J..

[30] Depienne C, LeGuern E (2012). PCDH19-related infantile epileptic encephalopathy: an unusual X-linked inheritance disorder. Hum Mutat..

[31] Goodman KM, et al. γ-Protocadherin structural diversity and functional implications. eLife. 2016;5:e20930. 10.7554/eLife.20930. 10.7554/eLife.20930 PMC5106212 27782885

[32] Dudek FE, Shao L-R (2003). Loss of GABAergic interneurons in seizure-induced epileptogenesis. Epilepsy Curr..

[33] Southwell DG (2012). Intrinsically determined cell death of developing cortical interneurons. Nature..

[34] Denaxa M (2018). Modulation of apoptosis controls inhibitory interneuron number in the cortex. Cell Rep..

[35] Wong FK (2018). Pyramidal cell regulation of interneuron survival sculpts cortical networks. Nature..

[36] Mancia Leon WR, et al. Clustered gamma-protocadherins regulate cortical interneuron programmed cell death. eLife. 2020;9:e55374. 10.7554/eLife.55374. 10.7554/eLife.55374 PMC7373431 32633719

[37] Mincheva-Tasheva S, Nieto Guil AF, Homan CC, Gecz J, Thomas PQ. Disrupted excitatory synaptic contacts and altered neuronal network activity underpins the neurological phenotype in PCDH19-clustering epilepsy (PCDH19-CE). Mol Neurobiol. 2021;58:2005–2018. 10.1007/s12035-020-02242-4. 10.1007/s12035-020-02242-4 33411240

[38] Dallosso AR (2012). Long-range epigenetic silencing of chromosome 5q31 protocadherins is involved in early and late stages of colorectal tumorigenesis through modulation of oncogenic pathways. Oncogene..

[39] Strong E (2015). Symmetrical dose-dependent DNA-methylation profiles in children with deletion or duplication of 7q11.23. Am J Hum Genet..

[40] El Hajj N (2016). Epigenetic dysregulation in the developing Down syndrome cortex. Epigenetics..

