# Supplementary material for: Biallelic variants in PCDHGC4 cause a novel neurodevelopmental syndrome with progressive microcephaly, seizures, and joint anomalies
Source: Genet Med. 2021 Jul 9;23(11):2138–49. doi: 10.1038/s41436-021-01260-4 (PMC8553613; doi:10.1038/s41436-021-01260-4)
Supplement: Supplementary file 1 — Supplementary Information [file 41436_2021_1260_MOESM1_ESM.pdf]

Supplementary Information

Fig. S1. Genome-wide linkage analysis of family 1. (a) LOD plot of parametric linkage analysis. Multipoint, LOD scores were calculated using MERLIN and a 100-k marker panel equally spaced by 10 kb. The highest LOD scores of 3.1 were obtained for a region of 8.9 Mb on chromosomes 5 and 6.9 Mb on chromosome 20, respectively. *PCDHGC4* is located in the identified interval on chromosome 5. (b) Haplotypes of generations IV and V of family 1 on chromosome 5. Haplotypes were reconstructed by MERLIN and presented graphically by HaploPainter. Marker IDs and genetic positons (cM) are given on the left. Markers located in the gene region of PCDHGC4 are boxed. Both affected subjects are homozygous for a region limited by the proximal and distal SNP markers rs2652080 and rs7716084, respectively.

a

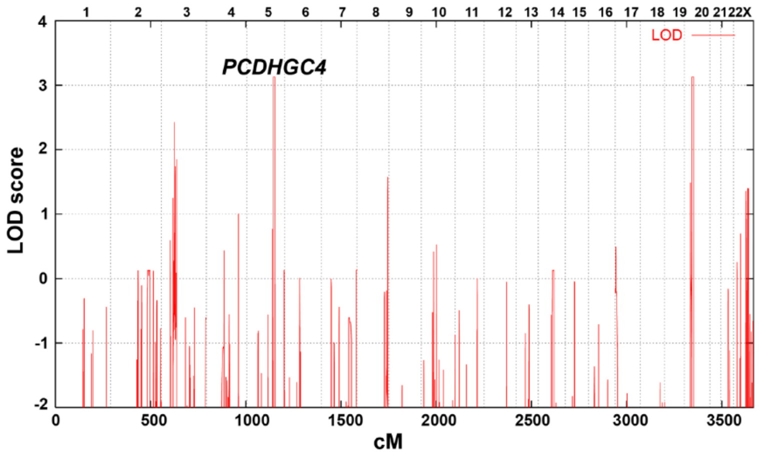

b

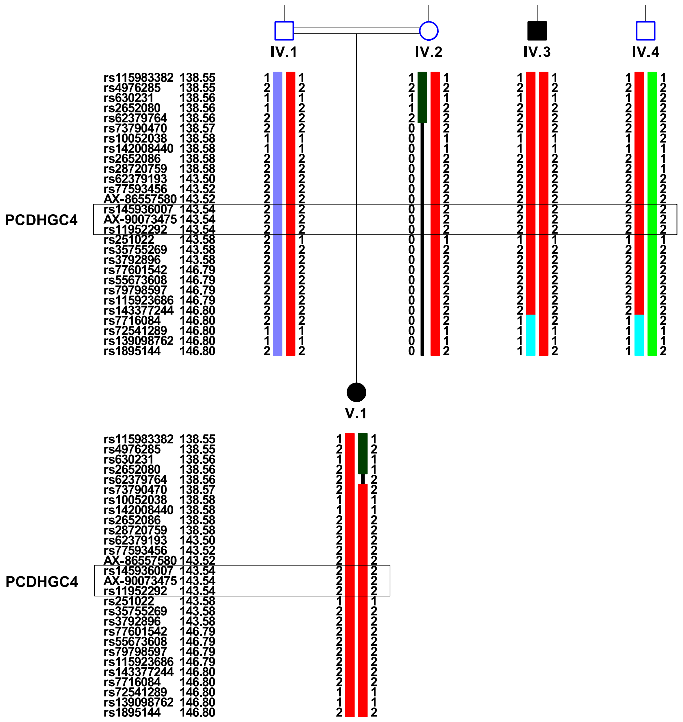

Fig. S2: Electropherograms of the identified variants in *PCDHGC4*. PCR and subsequent Sanger sequencing confirmed homozygosity of the identified *PCDHGC4* variants in all affected individuals and parental heterozygous status. Black arrows indicate positions of the altered nucleotides.

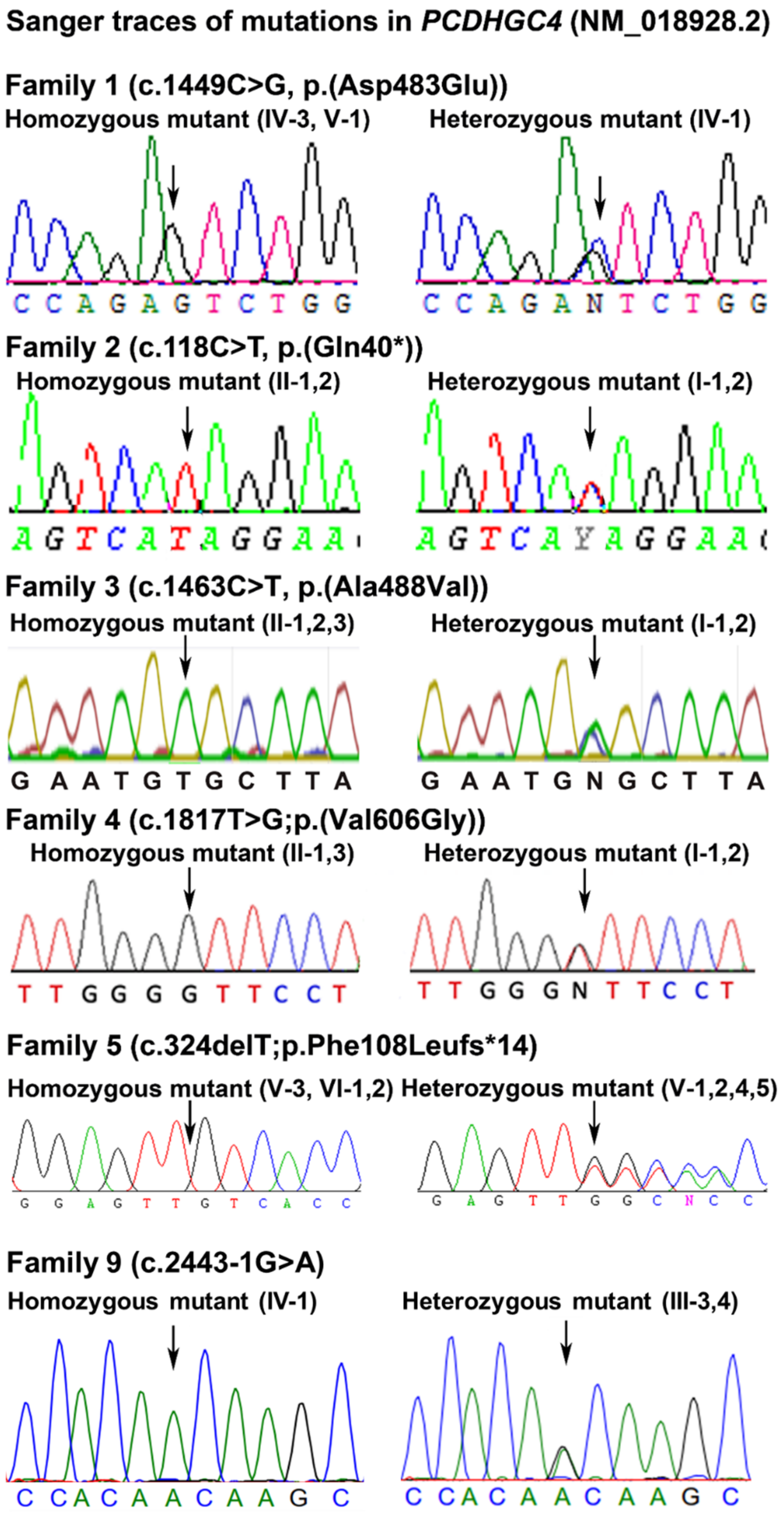

Fig. S3: Minigene splicing assay and sequencing results for the identified homozygous c.2443-1G>A variant in *PCDHGC4*. (a) Agarose gel electrophoresis of RT-PCR products showed two different transcripts for the identified sequence alteration G>A at position c.2443-1 of *PCDHGC4* (lane 3): one alternative product of smaller size, and one product of corresponding size compared to wild-type *PCDHGC4* exon 2 (Lane 1). Subsequent Sanger sequencing of the wild-type RT-PCR product revealed correct inclusion of *PCDHGC4* exon 2 (b), whereas the c.2443-1G>A variant cripples the endogenous splice-site and either leads to complete skipping of exon 2 or activates a cryptic, exonic acceptor splice-site (red asterisk) leading to the deletion of 4 bp (c.2443_2446del) and predicted to induce a frameshift and premature PCDHGC4 protein truncation (p.(Gln808Profs*42)) (c).

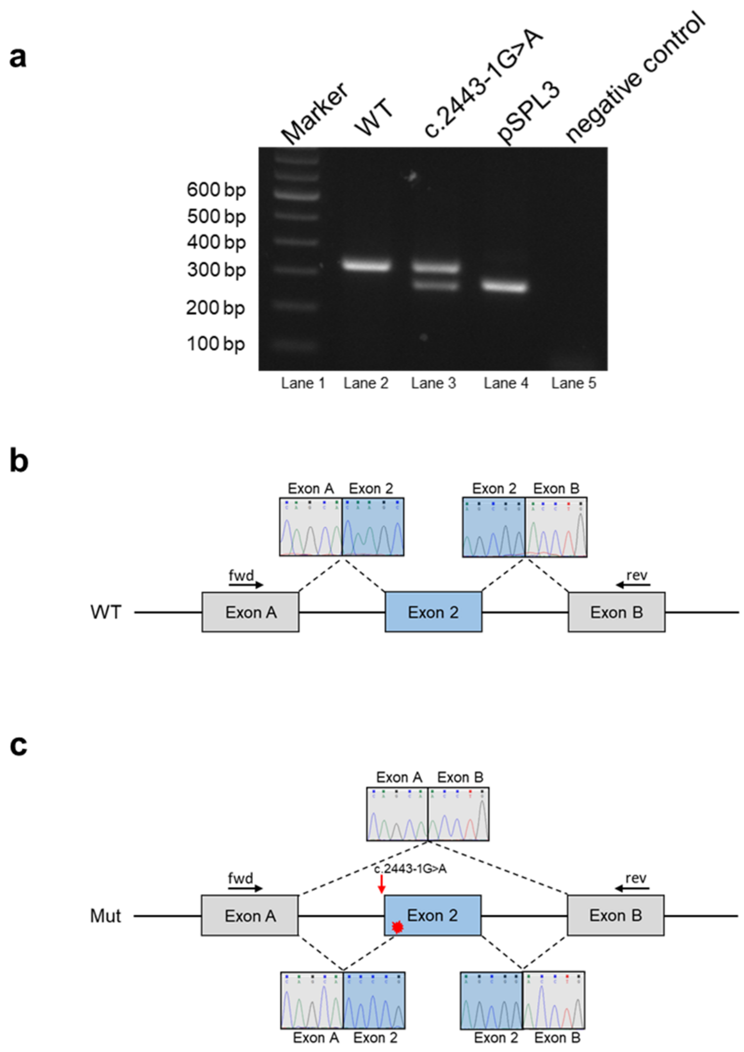

Fig. S4: Pedigree and clinical characteristics of individual V-1 (family S1) with the homozygous c.2524G>A (p.(Gly842Ser)) variant in *PCDHGC4*. (a) Pedigree of a consanguineous Iranian family. The affected individual V-1 (solid symbol) carries the c.2524G>A variant in *PCDHGC4* in a homozygous state while both non-affected parents are heterozygotes for this variant (IV-1 and IV-2, white symbols). (b) Facial features and hand anomaly observed in individual V-1. Clinical characteristics include a long philtrum and face, metopic craniosynostosis, ventriculomegaly and unilateral polydactyly (right hand). Additionally, we observed focal clonic seizures as well as moderate global developmental delay.

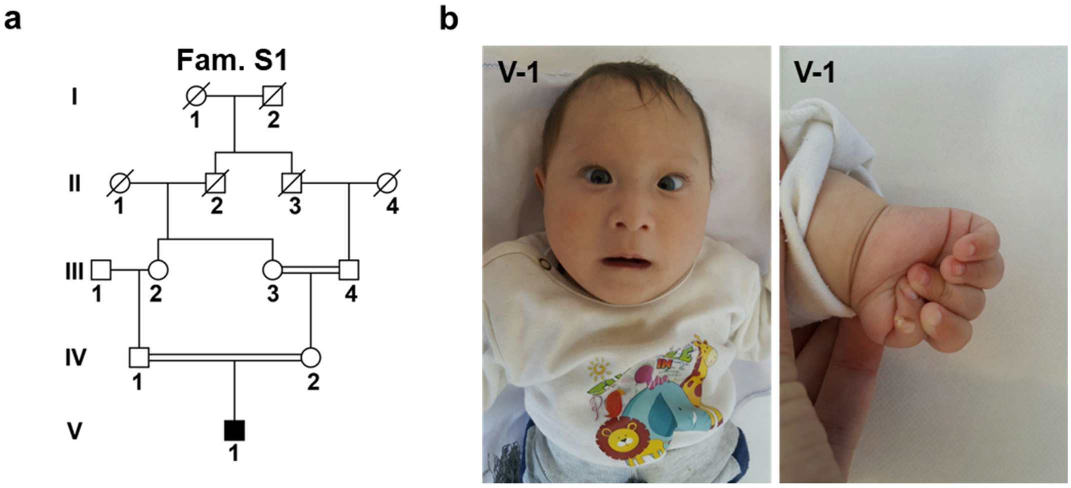

Clinical descriptions

Family 1

Subject IV-3 was the first-born child of healthy consanguineous parents of Pakistani origin. He was born at 38 weeks of gestation after an uneventful pregnancy. His birth weight was 3000 g, birth length and OFC were not recorded, but microcephaly was reported. He started walking at 4 years of age and showed gait abnormalities and ataxia. He had moderate intellectual disability and impaired speech development. At last evaluation at the age of 10 years, he presented with microcephaly (OFC = 45 cm, -4.7 SD) and short stature (127 cm, -2 SD). Neuroimaging revealed no structural brain anomalies, but showed a thin cerebral cortex. His maternal second nice, subject V-1, was born after 38 weeks of gestation and uneventful pregnancy. At birth, she weighed 2500 g. Signs of congenital microcephaly were reported, but OFC and birth length were not recorded. Similar to individual IV-3, gait abnormalities and ataxia were observed. She developed seizures and presented with severe intellectual disability. At her last evaluation at 8 years of age, she had developed short stature (113 cm, -2.5 SD) and microcephaly (OFC = 45 cm, -4.5 SD).

Family 2

Family 2 is a consanguineous family of Turkish origin. Subject II-1 was born at 40 weeks of gestation by uncomplicated spontaneous delivery as the first child of healthy parents after uneventful pregnancy. At birth, she had normal OFC (34 cm; -0.5 SD), weight (3850 g; 0.8 SD) and length (45 cm; -2.4 SD). She did not cry at birth and peripheral cyanosis was noted. She had hypotonia and developed neurodevelopmental and motor developmental delay and joint contractures. She walked independently at 32 months and had three seizures at the ages of 2, 5, and 7 years. EEG was normal and seizures were treated with antiepileptic drugs (carbamazepine) for 3 months. Brain MRI scan at 10 years showed cerebral atrophy. Puberty was normal. At 18 years of age, she had ataxic gait, hyperactivity attention deficit disorder, lumbar kyphoscoliosis, joint contractures, bilateral clinodactyly on both hands and hallux valgus. Extensive metabolic screening, conventional and molecular karyotyping were normal. At last evaluation at the age of 22 years, she presented with short stature

(153 cm, third centile) and microcephaly (OFC = 53 cm, third centile). She had moderate intellectual disability, was able to speak with a limited vocabulary, and had echolalia. She was able to recognize the letters of her name and could write in capital letters. She has a friendly behavior and face, and she is toilet trained. She attended a special needs school and swimming for skeletal deformity. Individual II-2, the sister of patient II-1, is a 13-year-old female born as the second child at 39 weeks of gestation after uneventful pregnancy. She was born via cesarean section due to decreased fetal movement. Her birth weight was 3500 g (0.5 SD), her length was 50 cm (0.2 SD), and she had a head circumference of 35 cm (0.6 SD). She had normal newborn scores and showed normal development. At the ages of 1.5 and 6.5 years she had non-febrile seizures without EEG abnormalities, which required no medical treatment. Brain MRI at 4 years of age was normal, and karyotyping and array-CGH revealed normal results. At her last physical examination at the age of 10, she presented with short stature (149 cm, <third centile), microcephaly (49 cm, <third centile), mild kyphosis, high narrow palate, high nasal bridge, joint contractures at elbows, and acral hypoplasia of toes. She had mild intellectual disability and poor communication skills due to lack of self-confidence. She was hyperactive and she had better speech and language development compared to her sister.

Family 3

Family 3 is a consanguineous family of Iraqi origin. Subject II-1 was born at term as the first child of healthy parents after an uneventful pregnancy. Birth weight and length were reported as within normal limits, and head circumference was not recorded. She presented with hypotonia at birth and was also noted to have broad thumbs. She showed global developmental delay and started walking between the age of 7 and 10 years. At the time of last examination at the age of 30 years, she presented with short stature (150 cm, -2.1 SD), microcephaly (51 cm, -2.5 SD) and hyperextensibility of small joints but otherwise non-dysmorphic. She was able to speak a few words and to dress with assistance. Her brother, subject II-2 in family 3, was the second-born child at term. Birth parameters including weight and length were also described as “within normal limits” and OFC was not recorded. He presented with similar clinical findings as his older sister including hypotonia at birth and broad

thumbs. He also had hypopigmented patches of the skin. He had global developmental delay and severe intellectual disability. He was nonverbal, but able to toilet and dress with assistance. At his last examination at the age of 27 years, he presented with mild growth restriction (164 cm, -1.8 SD) and microcephaly (51 cm, -3.7 SD). The youngest sibling of this family, subject II-3, presented at birth with a similar clinical presentation as her older siblings. Birth measurements were within limits and she had hypotonia. She started walking between the ages 7 and 10 and showed an unsteady gait. She had a single febrile seizure. At her evaluation at 24 years of age, she had developed short stature (150 cm, -2.1 SD) and microcephaly (49 cm, -3.9 SD). She presented with global developmental delay and severe intellectual disability.

Family 4

Subject II-1 is a 22-year-old female of Moroccan origin born as the first child of consanguineous healthy parents at 41 weeks of gestation. Her OFC was 34 cm (31st centile), her birth weight 3020 g (-1 SD) and her birth length was 45 cm (-3 SD). Neonatal adaptation was normal (APGAR score: 10-10). She had hypotonia, poor sucking reflex, and hypomobility with bilateral ulnar clubhand and valgus deformities. Unsupported sitting and babbling were acquired at 17 months. She walked independently at 24 months. First generalized seizures started at 5 years of age. Clinical examination at 5 and 9 years showed brisk deep tendon reflexes and mild ataxia. Brain MRI scan at the age of 17 years showed mild cerebral atrophy. Extensive metabolic screening as well as puberty were normal. At last evaluation at the age of 22 years, she presented with short stature (152 cm; -2 SD) and microcephaly (OFC = 52 cm, -2 SD). Her weight was 48 kg and she had a lumbar scoliosis. She presented with mild facial dysmorphic features including a long face, round ears with attached lobes, thick lips with everted inferior lips, and mild prognathism. She had moderate intellectual disability and was able to speak with a relatively good vocabulary, but also had echolalia. She recognized the letters of her name, knew the alphabet, and could copy a little text in capital letters. She was toilet trained. She had relatively good autonomy as she could stay at home and could go to well-known places on her own. She attended a special needs school. Epileptic remission was achieved with three antiepileptic drugs (phenobarbital, lamotrigine,

and sodium valproate). Her sister, subject II-2, presented at birth with a similar clinical presentation including global hypotonia and bilateral ulnar clubhand. At the age of 18 months, she was able to sit independently, but did not walk or show any speech development. At that age, she died due to *status epilepticus* triggered by fever of unknown origin. Her brother, the third-born child (II-3), was born at 41 weeks of gestation with normal OFC (34 cm; -1 SD) and weight (3300 g; 23.5th centile). His birth length was 48 cm (-2 SD). He also had hypotonia and deformities including bilateral ulnar clubhand, flexion contracture of his legs, and clubfoot in line with the clinical presentation of *arthrogryposis multiplex congenita*. He sat independently at 12 months of age, crawled at 16 months, started babbling at 18 months, and walked at 10 years. First generalized seizures started at 18 months. At that time, clinical examination showed normal central muscle tone, peripheral hypertonia with brisk deep tendon reflexes, and joint stiffness. Brain MRI scan at 7 years of age showed cerebral atrophy. Extensive metabolic screening as well as abdominal ultrasound was normal. At last evaluation at the age of 14 years, he presented with short stature (height = 138 cm, -2 SD) and microcephaly (OFC = 51 cm, -2 SD). His weight was 38 kg. He presented with similar facial dysmorphic features as his older sister including a long face, round ears with attached lobes, thick lips with everted inferior lips, and mild prognathism. He had severe intellectual disability with absent speech, but he was learning pictorial language. He was able to help undressing and still had bedwetting. He attended a special needs school. He had epilepsy remission with two antiepileptic drugs, clobazam and levetiracetam.

Family 7

Family 7 include five siblings from first cousin Lebanese parents. Three of the five siblings at the ages of 17, seven and two years share a common neurocognitive disorder including neurological, growth and ocular features (Fig. 1). The oldest boy, individual III-1, was born at term after an uncomplicated pregnancy. In the newborn period he was noted to have markedly increased tone with an exaggerated startle response and a tendency to adopt a rigid flexed posture. He was able to latch and feed in the newborn period and over the first 12 months, his increased tone gradually improved, but he has been left with persistent hypertonia and some restriction of extension most notable at his elbows. He was

noted to have a problem with ocular movement and was diagnosed with an oculomotor apraxia. He had delayed motor milestones including not walking until after three years of age, and a tendency to toe walk was observed. An Achilles tendon release and ankle orthoses from the third year of life improved mobility. He has a moderate global intellectual disability and has been educated in a school for children with intellectual and physical difficulties. He has good receptive and functional expressive language with some articulation difficulties. He has minimal literacy skills. He has basic self-help skills including dressing, showering and toileting with assistance. His growth slowed at around the time of puberty and he now has short stature (-2.9 SD). He has had multiple investigations including chromosome array, fragile X testing, metabolic testing including urine metabolic screens, CSF lactate and amino acids, lysosomal enzymes, transferrin isoforms, VLCF and blood lactate. He has had a normal EEG, nerve conduction and EMG studies. Sequential MRIs have shown some thinning of the anterior corpus collosum and mild ventriculomegaly, and increase in the extra axial spaces. He also has a large temporal fossa arachnoid cyst. Physically his most recent height was 155.5cm (-2.9 SD), weight was 72kg (+0.4 SD) and head circumference was 56cm (-0.1 SD). He had dysmorphic facial features with full arched eyebrows and synophyrus, a high nasal bridge, hooked nose and overhanging columella, short philtrum and full lips with a narrow high palate. He had some malar flattening and micrognathia. Peripherally persisting fetal fingertip pads, and mild 5th finger clinodactyly. There was some restriction of extension of his elbows. He had some wasting of the forearms and lower limbs with reduced muscle bulk. Power was normal and reflexes brisk and showed spreading. There was no facial weakness. Ocular tracking was absent. The younger affected brother, individual III-4, presented with an identical clinical pattern with stronger motor skills walking at two years and three months. He required an Achilles tendon release in the second year of life and now has no significant joint restriction. He still has no clear single words at age seven, but better receptive language. He is not toilet trained. He has a moderate to severe global developmental delay. He also has an oculomotor apraxia, MRI changes including thinning of the corpus collosum and mild ventricular enlargement. Physically his most recent height was 155.5cm (-2.9 DS), weight was 72 kg (+0.4 SD) and head circumference was 56cm (-0.1 SD) At his last evaluation, he had normal growth parameters (height 126 cm (+0.4 SD), weight 21 kg, head

circumference 51 cm (-0.9 SD). The youngest affected brother, individual III.5, shares identical features. He walked at two years and two months, and his tone and reflexes have now normalised with no residual joint restriction. He is babbling but has no clear words, and was also diagnosed with an oculomotor apraxia. At last evaluation, he his growth parameters were: height = 95 cm (-1.3 SD), OFC = 47 cm (-1.2 SD), and weight = 13 kg.

Family 8

Individual IV-I (Fig. 1) is the first child born to multiple consanguineous Sudanese parents who had previously experienced two miscarriages. She was born at term by normal vaginal delivery after prolonged rupture of membranes lasting 4 days. She displayed respiratory distress at birth requiring admission to NICU and was treated with antibiotics. Head circumference and weight at birth were 32.4 cm (-1.25 SD) and 2330 g (-2.17 SD), respectively. She was noted to have micrognathia, overlapping fingers on the right hand, elbow contractures and rocker-bottom feet with valgus position of her feet and hands. A clinical diagnosis of Arthrogryposis Multiplex Congentia was made and she underwent Achilles tendon lengthening procedure whilst in Sudan at the age of 5 years. She has also been listed for left elbow release in the near future. She has oculomotor apraxia with saccadic initiation failure. She has had four febrile convulsions, two of which required benzodiazepines treatment. Since the age of 5 years, however, she has had no further seizures and has not required anti-epileptic treatment. She began walking at 5 years 4 months but her speech remains limited. Her most recent growth parameters were documented aged 6 years 6 months with weight 15.1 kg (-2.90 SD), height 104 cm (-2.87 SD) and OFC 46.5 cm (-3.72 SD). At last clinic review aged 8 years 5 months, she had no clear speech but was able to copy some words and follow simple commands. An MRI brain aged 6 years showed mildly prominent frontal horns with mild atrophy but no other structural brain abnormality. Plasma amino acids and carnitine profile were normal, as well as very long chain fatty acids and TORCH screen. A microarray and Fragile X testing were both normal.

Supplemental Material and Methods

Subjects

All subjects or their legal representatives gave written informed consent for the molecular genetic analyses, for publication of the photographs and the results. DNA from participating family members was extracted from peripheral blood lymphocytes by standard extraction procedures.

Genome-wide linkage analysis

Genome-wide linkage analysis in family 1 was performed using the Axiom Precision Medicine Research Array (Affymetrix) according to manufacturer's instructions. Subsequent data handling was performed using the graphical user interface ALOHOMORA.[1] Relationship errors were identified using the program Graphical Relationship Representation.[2] The program PedCheck was applied to find Mendelian errors and data for SNPs with such errors were removed from the data set.[3] Non-Mendelian errors were identified using the program MERLIN and unlikely genotypes for related samples were deleted.[4] For genotyping, DNA samples of two affected individuals (IV-3 and V-1), their parents (III-5, III-6, and IV-1) and one healthy sibling (IV-4) of family 1 were used assuming an autosomal recessive inheritance pattern, full penetrance and a disease allele frequency of 0.0001. Multipoint LOD scores were calculated using the program MERLIN.[4] Haplotypes were constructed with MERLIN and presented graphically with HaploPainter.[4,5]

Genome/exome sequencing and filtering strategies

Exome sequencing (ES) of individual IV-3 (family 1) was carried out using the Agilent SureSelect V6 (Agilent) enrichment kit on an Illumina HiSeq4000 as previously described.[6,7] ES data analysis and filtering of mapped target sequences was performed using the 'Varbank2' exome analysis pipeline of the Cologne Center for Genomics (CCG, University of Cologne, Germany).[8] In family 1, we performed genome-wide linkage analysis assuming an autosomal recessive inheritance pattern with full penetrance and a disease allele frequency of 0.0001. Genotyping of DNAs of both affected (Fig. 1a, IV-

3 and V-1) and five healthy individuals (Fig. 1a, II-5, III-6, IV-1, IV-2, and IV-4) of family 1 identified two regions with a maximum possible LOD score of 3.1 on chromosomes 5 and 20 (Fig. S1). These homozygous regions encompass 8.86 Mb on chromosome 5 (5:135,277,003 to 5:144,145,201) and 6.90 Mb on chromosome 20 (20:17,630,754 to 20:24,535,473). We performed ES on DNA of individual IV-3 and identified a single rare, homozygous variant located within these two chromosomal homozygous regions predicted to have a severe impact on the corresponding protein function. This variant, c.1449C>G, is located in exon 1 of *PCDHGC4* and predicted to lead to the substitution of an aspartate at the amino acid position 483 with glutamate (p.(Asp483Glu)). Subsequent Sanger sequencing confirmed the presence of this variant in individual IV-3 and co-segregation analysis revealed homozygosity for this variant in the affected individual V-1 as well as heterozygous status for the variant in the healthy parents of both affected individuals (III-5,III-6, IV-1 and IV-2).

In family 2, ES of both affected children was carried out using the IDT xGen Exome Research Panel v1.0 enrichment kit (Integrated DNA Technologies) on an Illumina NovaSeq6000. ES data analysis and filtering of variants were carried out using the exome analysis pipeline 'Varvis' (Limbus Medical Technologies GmbH), and we obtained a 20fold coverage in >94% of target sequences. We excluded homozygous or compound heterozygous variants in any of the OMIM-referenced genes that are associated with autosomal recessive isolated or syndromic forms of microcephaly, and we analyzed the ES data for variants with a coverage of more than 6 reads, an allele frequency ≥75% in both affected individuals, and a minor allele frequency (MAF) <0.5% in the gnomAD database. Applying these filter criteria, we identified in total 8 shared homozygous variants, 7 of them inducing a single amino acid substitution predicted to have minor to no influence on the encoded protein by different *in silico* prediction programs. Only a single homozygous variant was predicted to have a severe impact on protein function. Both affected individuals carried the homozygous variant c.118C>T in *PCDHGC4* that induces the formation of a premature stop codon at the amino acid position 40 (p.(Gln40*)) (Fig. S2).

In family 3, exome capture and high-throughput sequencing of genomic DNA was performed for two of the three affected siblings (subjects II-2 and II-3). Targeted exon capture was done using the Agilent

SureSelect All Exon 50 MB (V5) exome enrichment kit and sequencing was performed with an Illumina HiSeq2000 using 2x100bp chemistry. Read alignment, variant calling, and annotation were done as outlined for previous FORGE and Care4Rare projects.[9,10] with a pipeline based on Burrows-Wheeler Aligner (BWA),[11] Picard (http://picard.sourceforge.net/), ANNOVAR,[12] and custom annotation scripts. Analyses were performed under recessive modes of inheritance based on the family history. The variants were prioritized by allele frequency (less than 1% in our local Care4Rare database). Only a single homozygous variant detected in both affected siblings was predicted to have severe impact on protein function based on *in silico* prediction programs and was absent in the homozygous state in gnomAD and the local Care4Rare database. Both affected individuals carried the homozygous variant c.1463C>T in *PCDHGC4* that results in the substitution at p.(Ala488Val). Sanger sequencing confirmed homozygosity for this variant in both affected individuals as well as their affected sibling II-1. The healthy father was heterozygous for this variant.

ES of family 4 (subjects I-2, II-1 and II-3) was performed using the NimbleGen SeqCap EZ MedExome sequencing kit (Roche) following standard protocols. Paired-end 2x75 bp sequencing was performed on a NextSeq500 sequencer (Illumina). Sequencing data were analyzed according to an autosomal recessive inheritance pattern, and variants were selected based on their MAF and their effect on protein coding sequence. We filtered for variants with a MAF <0.1% in the gnomAD database, present in both affected individuals and predicted to be pathogenic or likely pathogenic by at least three out of five *in silico* prediction programs. Resulting variants were filtered based on expression of encoded proteins using the Genotype-Tissue Expression (GTex) portal. This analysis revealed a single homozygous variant in *PCDHGC4*. Both affected individuals carried the homozygous variant c.1817C>T in *PCDHGC4*. On protein level, this variant leads to the substitution of a valine at the amino acid position 606 with glycine (p.(Val606Gly)). We confirmed this variant by Sanger sequencing and co-segregation analysis revealed heterozygous status of both parents (Fig. S2).

In families 5 and S1, ES on each proband was performed at Macrogen (Korea). In brief, target enrichment was performed with 2 μg genomic DNA using the SureSelectXT Human All Exon Kit version

6 (Agilent Technologies) to generate barcoded ES libraries. Libraries were sequenced on the HiSeqX platform (Illumina) with 50x coverage. Quality assessment of the sequence reads was performed by generating QC statistics with FastQC (http://www.bioinformatics.bbsrc.ac.uk/projects/fastqc). ES data was filtered for exonic and donor/acceptor splicing variants. In accordance with the pedigree and phenotype, priority was given to rare variants (<0.01% in public databases, including 1,000 Genomes project, NHLBI Exome Variant Server, Complete Genomics 69, and Exome Aggregation Consortium [ExAC v0.2]) that were fitting a recessive (homozygous or compound-heterozygous) or a de novo model and/or variants in genes previously linked to congenital microcephaly, developmental delay and other neurological disorders. Identified *PCDHGC4* variants, c.324del (p.(Phe108Leufs*14)) in family 5, and c.2524G>A (p.(Gly842Ser)) in family S1, were confirmed by Sanger sequencing and tested for co-segregation analysis in the respective families (Fig. S2).

In family 8, genome sequencing (GS) of parent-patient trio was performed in the course of the 100,000 genomes project (UK) on a HiSeqX sequencer (Illumina).[13] Initial filtering employed a 1 % population allele frequency threshold, and variants were analyzed using the LabKey application available within Genomic England's research environment.

In family 9, ES on DNA samples of the affected individual IV-1 was performed as previously described.[14] In brief, exome capture was performed using the SureSelect V6 (Agilent) kit following manufacturer's instructions. Sequencing was carried out on a HiSeq4000 sequencer (Illumina). Data analysis was performed a pipeline based on Burrows-Wheeler Aligner (BWA),[11] Picard (http://picard.sourceforge.net/), GATK, and SnpEff. The variants were filtered based on coverage (> 10x), quality (Phred score ≥ 30), and MAF (≤ 0.005) in the 1000 Genomes Project and EVS6500. Variants were further characterized based on their coding effect (non-synonymous, indels, and splice-site variants), and artifact-prone genes were excluded. Missense variants were analyzed by using a combination of *in silico* prediction tools including CADD, MutationTaster, PolyPhen-2, and SIFT. Variants with potential effects on splicing were characterized using ESEfinder and RESCUE-ESE.[15,16] This analysis revealed the homozygous variant c.2443-1G>A in *PCDHGC4* in the affected individual IV-1

affecting the invariant acceptor splice-site of intron 1 of *PCDHGC4*. We confirmed this variant by Sanger sequencing and co-segregation analysis revealed heterozygous status of both parents (Fig. S2).

Minigene Assay / pSPL3 splicing assay

In vitro analysis of the potential splice-site variant c.2443-1G>A was performed using the pSPL3 splicing assay. A 929 bp genomic region including exon 2 (59 bp) and flanking introns (360 bp upstream and 510 bp downstream of exon 2) was PCR amplified from genomic DNA of a control individual and individual IV-1 (family 9) with primers containing *Xho*I and *Bam*HI restriction site (forward primer with *Xho*I restriction site: 5'-aattctcgagGCTTTGTCTGCACCTCTTCC-3' and reverse primer with *Bam*HI restriction site: 5'- attggatccTTCCTCAGTTCCACCTCCAG-3'). PCR fragment ligation in a linearized pSPL3-vector preceded transformation into DH5α competent cells (NEB 5-alpha, New England Biolabs) and overnight incubation. The wild-type and mutant-containing vector sequences were confirmed by Sanger sequencing and transfected into HEK293T cells using the FuGENE 6 Transfection Reagent (Promega). Empty vector and transfection negative reactions controls were included. Total RNA was prepared from 24-hour post-transfected cells using a miRNAeasy Mini Kit (Qiagen), and reverse transcription was carried out using a High Capacity RNA-to-cDNA Kit (Applied Biosystems). Amplified fragments were visualized on a 2% agarose gel, cloned using the TA cloning dual promoter with pCRII kit (Invitrogen), and subsequently Sanger sequenced.

Web Resources

Clustal Omega, https://www.ebi.ac.uk/Tools/msa/clustalo/

Database of Genomic Variants (DGV), http://dgv.tcag.ca/

DECIPHER database, https://decipher.sanger.ac.uk/

Ensembl, https://www.ensembl.org/index.html

GTEx portal: https://www.gtexportal.org/home/

gnomAD browser, https://gnomad.broadinstitute.org/

Mutalyzer, https://mutalyzer.nl/

MutationTaster, https://www.mutationtaster.org

OMIM, https://www.ncbi.nlm.nih.gov/omim

PolyPhen-2, https://genetics.bwh.harvard.edu/pph2/

Protein Data Bank, www.wwpdb.org

SIFT, https://sift.bii.a-star.edu.sg/

UCSC genome browser, https://genome.ucsc.edu/

UniProtKB, https://www.uniprot.org/

Varbank2, https://varbank.ccg.uni-koeln.de/varbank2/

Supplemental References

1. Rüschendorf F, Nürnberg P. ALOHOMORA: a tool for linkage analysis using 10K SNP array data. *Bioinformatics*. 2005;21(9):2123-2125. doi:10.1093/bioinformatics/bti264

2. Abecasis GR, Cherny SS, Cookson WO, Cardon LR. GRR: graphical representation of relationship errors. *Bioinformatics*. 2001;17(8):742-743. doi:10.1093/bioinformatics/17.8.742

3. O'Connell JR, Weeks DE. PedCheck: a program for identification of genotype incompatibilities in linkage analysis. *Am J Hum Genet*. 1998;63(1):259-266. doi:10.1086/301904

4. Abecasis GR, Cherny SS, Cookson WO, Cardon LR. Merlin--rapid analysis of dense genetic maps using sparse gene flow trees. *Nat Genet*. 2002;30(1):97-101. doi:10.1038/ng786

5. Thiele H, Nürnberg P. HaploPainter: a tool for drawing pedigrees with complex haplotypes. *Bioinformatics*. 2005;21(8):1730-1732. doi:10.1093/bioinformatics/bth488

6. Hussain MS, Baig SM, Neumann S, et al. A truncating mutation of CEP135 causes primary microcephaly and disturbed centrosomal function. *Am J Hum Genet*. 2012;90(5):871-878. doi:10.1016/j.ajhg.2012.03.016

7. Hussain MS, Baig SM, Neumann S, et al. CDK6 associates with the centrosome during mitosis and is mutated in a large Pakistani family with primary microcephaly. *Hum Mol Genet*. 2013;22(25):5199-5214. doi:10.1093/hmg/ddt374

8. Szczepanski S, Hussain MS, Sur I, et al. A novel homozygous splicing mutation of CASC5 causes primary microcephaly in a large Pakistani family. *Hum Genet*. 2016;135(2):157-170. doi:10.1007/s00439-015-1619-5

9. Srour M, Schwartzentruber J, Hamdan FF, et al. Mutations in C5ORF42 cause Joubert syndrome in the French Canadian population. *Am J Hum Genet*. 2012;90(4):693-700. doi:10.1016/j.ajhg.2012.02.011

10. Beaulieu CL, Majewski J, Schwartzentruber J, et al. FORGE Canada Consortium: outcomes of a 2-year national rare-disease gene-discovery project. *Am J Hum Genet*. 2014;94(6):809-817. doi:10.1016/j.ajhg.2014.05.003

11. Li H, Durbin R. Fast and accurate short read alignment with Burrows-Wheeler transform. *Bioinformatics*. 2009;25(14):1754-1760. doi:10.1093/bioinformatics/btp324

12. Wang K, Li M, Hakonarson H. ANNOVAR: functional annotation of genetic variants from high-throughput sequencing data. *Nucleic Acids Res*. 2010;38(16):e164. doi:10.1093/nar/gkq603

13. Turnbull C, Scott RH, Thomas E, et al. The 100 000 Genomes Project: bringing whole genome sequencing to the NHS. *BMJ*. 2018;361:k1687. doi:10.1136/bmj.k1687

14. Vona B, Mazaheri N, Lin S-J, et al. Biallelic mutation of CLRN2 causes non-syndromic hearing loss in humans. *bioRxiv*. Published online September 1, 2020:2020.07.29.222828. doi:10.1101/2020.07.29.222828

15. Cartegni L, Wang J, Zhu Z, Zhang MQ, Krainer AR. ESEfinder: A web resource to identify exonic splicing enhancers. *Nucleic Acids Res*. 2003;31(13):3568-3571. doi:10.1093/nar/gkg616

16. Fairbrother WG, Yeo GW, Yeh R, et al. RESCUE-ESE identifies candidate exonic splicing enhancers in vertebrate exons. *Nucleic Acids Res*. 2004;32(Web Server issue):W187-190. doi:10.1093/nar/gkh393
